# Catalytic Performance of CuZnAl Hydrotalcite-Derived Materials in the Continuous-Flow Chemoselective Hydrogenation of 2-Methyl-2-pentanal toward Fine Chemicals and Pharmaceutical Intermediates

**DOI:** 10.3390/molecules29143345

**Published:** 2024-07-16

**Authors:** Rahma Abid, Bartosz Zawadzki, Jaroslav Kocik, Grzegorz Słowik, Janusz Ryczkowski, Mirosław Krawczyk, Zbigniew Kaszkur, Izabela S. Pieta, Anna Śrębowata

**Affiliations:** 1 Institute of Physical Chemistry, Polish Academy of Sciences, ul. Kasprzaka 44/52 PL, 01-224 Warsaw, Poland; bzawadzki394@gmail.com (B.Z.); mkrawczyk@ichf.edu.pl (M.K.); zkaszkur@ichf.edu.pl (Z.K.); ipieta@ichf.edu.pl (I.S.P.); 2 ORLEN UniCRE a.s., Záluží 1, 436 70 Litvínov, Czech Republic; jaroslav.kocik@orlenunicre.cz; 3 Department of Chemical Technology, Faculty of Chemistry, Maria Curie-Skłodowska University, Plac Maria Curie-Skłodowskiej 3, 20-031 Lublin, Poland; grzesiek.slowik@gmail.com (G.S.); janusz.ryczkowski@mail.umcs.pl (J.R.)

**Keywords:** porous hydrotalcite-derived materials, 2-methyl-2-pentenal chemoselective hydrogenation, 2-methyl-2-penten-1-ol, 2-methylpentanal

## Abstract

Hydrotalcite-derived materials are eco-friendly, cheap, and efficient catalysts of different reactions. However, their application in liquid-phase hydrogenation could be more extensive. Hence, this work concerns the application of three hydrotalcite-derived materials with different CuZnAl molar ratios in the liquid-phase continuous-flow hydrogenation of 2-methyl-2-pentenal (MPEA) at a wide range of temperature (298–378 K) and pressure (1 × 10^6^–6 × 10^6^ Pa). The catalytic investigations were supported by catalysts characterization by ICP-OES, TPR, in situ XRD, XPS, NH_3_-TPD, CO_2_-TPD, and TEM measurements on different stages of their biography. It was shown that the catalytic activity of these samples is related to the Cu^0^/Cu^+^ ratio. Depending on the reaction conditions, selectivity control is possible. All catalysts were 100% selective to 2-methylpentanal (MPAA)—sedative drug precursor, with low conversion, at temperatures ≤ 338 K at every pressure. However, the selectivity of the second desired product, fragrance intermediate, 2-methyl-2-penten-1-ol (MPEO), increased significantly at higher temperatures and pressures. It reached the unique value of 54% with 60% substrate conversion at 378 K and 6 × 10^6^ Pa for the catalyst with the highest Cu loading. It was revealed that the production of significant amounts of MPEO is related to the reaction conditions, the Cu^+^ predominance on the surface, the hydrogen spillover effect, and the acid–base properties of these systems.

## 1. Introduction

Heterogeneous catalysis is an essential important field in fine chemistry chemical synthesis. In this very demanding area, chemoselective hydrogenation of α,β-unsaturated aldehydes into unsaturated alcohols and saturated aldehydes, crucial in producing fragrances, flavors, and pharmaceuticals, plays an important role [1,2,3,4]. An example of the α,β-unsaturated aldehyde is 2-methyl-2-pentenal (MPEA). The chemoselective hydrogenation of the C=O bond in the MPEA molecule leads to the formation of 2-methyl-2-penten-1-ol (MPEO). This alcohol has potential in fragrance synthesis [5]. On the other hand, the hydrogenation C=C in MPEA leads to the formation of 2-methylpentanal (MPAA), which is used in the fabrication of drugs, among them meprobamate, which belongs to the anxiolytic class [6].

**Scheme 1:**
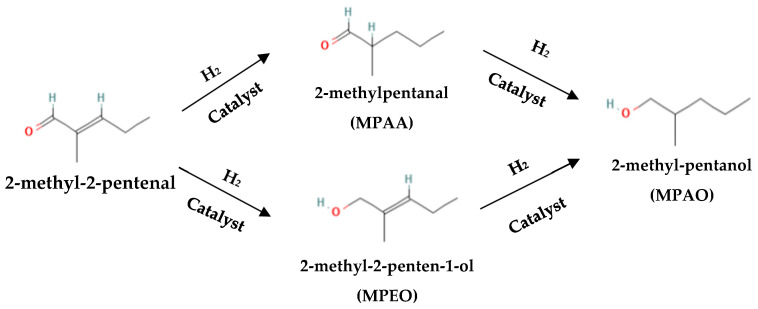
Hydrogenation of 2-methyl-2-pentenal.

A thorough analysis of the literature showed that only a few works have carried out the hydrogenation of 2-methyl-2-pentenal (MPEA) [7,8,9]. T.T. Pham et al. [9] investigated the gas-phase conversion of MPEA with Pd, Pt, and Cu supported on silica at 473 K. All the catalysts were active for both C=O and C=C bonds. However, 0.5 wt.% of Pd and Pt/SiO_2_ catalysts were more selective to C=C. On the other hand, 5 wt.% Cu/SiO_2_ was more selective to C=O producing primarily MPEO at very low conversion. At high conversion, the situation changed; MPEO was converted to undesired 2-methyl-pentanol (MPAO) in equilibrium with MPAA (Scheme 1).

However, the application of active carbon supported 6 wt.% Cu in the liquid-phase continuous-flow MPEA hydrogenation leads to the formation of 100% of 2-methylpentanal at 318 K and 6 × 10^6^ Pa with 81% of substrate conversion [10]. This phenomenon was explained by the metal–support interaction. The electron transfer from the active carbon to copper leads to the formation of Cu^0^, which shows a higher affinity to C=C than C=O. Furthermore, the chemoselective hydrogenation of MPEA with Co supported on active carbon showed that the best catalyst with high TOF and selectivity to MPAA is also 6 wt.% Co, which has more active sites to hydrogenate the C=C bond in the liquid-phase continuous-flow conditions [11]. However, obtaining unsaturated alcohol with high selectivity remains a challenge.

An attractive alternative for typical metal/support systems in chemoselective hydrogenation could be hydrotalcite (HT) or layered double hydroxide (LDH)-derived materials. Most of HT and LDH are synthetic, relatively simple, and cheap to prepare on both a laboratory and industrial scale [12,13]. Their properties, like the anion-exchange capacity of the interlayer region, the adsorption capacity, the adjustable surface basicity, and the cation-exchange capacity of the brucite layer, give them a chance to create an attractive alternative for typical metal/support systems [14,15]. In addition, HT and LDH-type materials are recognized as providing eco-friendly and low-cost solutions for problems related to pollution or energy requirements [16,17,18]. J. Marchi et al. [19] studied the effect of the structure of Cu-based catalysts in the hydrogenation of cinnamaldehyde (3-phenylprop-2-enal, CNA) in the liquid phase. They showed that ternary Cu–Zn–Al and quaternary Cu–Ni (Co)–Zn catalysts are more active than Cu/SiO_2_ and produce predominantly cinnamyl alcohol. Dragoi et al. [20] investigated the catalytic performance of ZnAl LDH with various amounts of Cu and Ni atoms in liquid-phase hydrogenation of cinnamaldehyde. This study showed that the formation of cinnamyl alcohol increased on spinel Cu-Ni (Co)-Zn-Al due to the formation of Cu^0^-M^2+^ dual sites. Rudolf et al. [21] performed the catalytic activities of LDH with Ni or Co incorporated in the brucite-like layers, besides Al, in the chemoselective hydrogenation of cinnamaldehyde. This study revealed that the Co-Al catalyst is less active than Ni-Al. On the other way, Ni-Al is more selective to the C=C bond, which produces (~95% selectivity to hydrocinnamaldehyde at complete conversion of cinnamaldehyde). Co-Al is more selective to the C=O bond to obtain ~60% of cinnamyl alcohol at ~50% conversion of cinnamaldehyde. 

These results were good prognostic and motivation for the investigations of CuZnAl hydrotalcite-derived materials with different Cu loadings in the liquid-phase continuous-flow chemoselective hydrogenation of α,β-unsaturated aldehyde (2-methyl-2-pentenal).

## 2. Results and Discussion

### 2.1. Inductively Coupled Plasma-Optical Emission Spectroscopy (ICP-OES) Results

The ICP-OES technique examined whether the obtained ratio of metals in the catalysts corresponds to the theoretical one. All catalysts showed Cu and Zn content slightly higher but not significantly higher than expected. The molar composition of the metals in CuZnAl is (0.61:1.26:1), (1.23:1.26:1), and (2.28:1.12:1) for the three samples, respectively. These materials will be marked as Cu**_0.61_**Zn**_1.26_**Al**_1_**, Cu**_1.23_**Zn**_1.26_**Al**_1_**, and Cu**_2.28_**Zn**_1.12_**Al**_1_**. 

### 2.2. Temperature-Programmed Reduction (H_2_-TPR)

**Figure 1:**
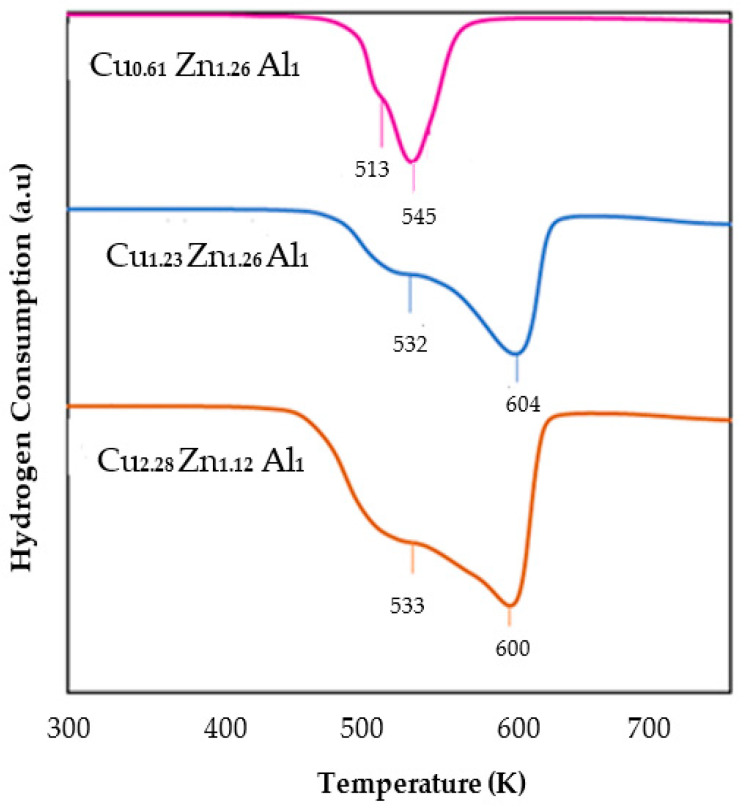
Temperature-programmed reduction of the CuZnAl mixed oxides.

Temperature-programmed reduction in mixed metal oxides was conducted to determine the appropriate reduction temperature for CuZnAl materials. For all three catalysts, two distinct but not fully separated peaks can be seen (Figure 1). 

As shown in Figure 1, the reduction profiles of all samples show a broad two-shoulder peak with a reduction maximum in the temperature range 450–635 K. This phenomenon can be attributed to the reduction in two different types of CuO phases: dispersed CuO reduced at low temperature (~532 K) and larger CuO clusters reduced at high temperature (~600 K) [22,23,24]. For Cu**_0.61_**Zn**_1.26_**Al**_1_**, the peak at a lower temperature (513 K) can be attributed to the reduction of highly dispersed CuO, related to the more straightforward reduction of part of the metallic phase for the sample with the lowest copper content [22]. However, TPR profiles for Cu**_1.23_**Zn**_1.26_**Al**_1_** and Cu**_2.28_**Zn**_1.12_**Al**_1_** systems are almost identical. They differ only in signal intensity, an understandable phenomenon resulting from the increased copper content in the material. The higher reduction temperature of Cu**_1.23_**Zn**_1.26_**Al**_1_** and Cu**_2.28_**Zn**_1.12_**Al**_1_** systems compared to Cu**_0.61_**Zn**_1.26_**Al**_1_** may indicate a stronger interaction of CuO with the support. The presence of two TPR peaks may be the effect of subsequent reduction from Cu^2+^ to Cu^+^ and Cu^+^ to Cu^0^, as was observed earlier [25]. 

The optimal reduction temperature of CuZnAl mixed oxides was slightly higher than determined by the TPR measurements to guarantee the metal precursor’s total reduction to the metallic form. The CuZnAl materials were reduced for 3 h at 673 K in a 10% H_2_/Ar mixture before characterization and catalytic run.

### 2.3. In Situ X-ray Diffraction

**Figure 2:**
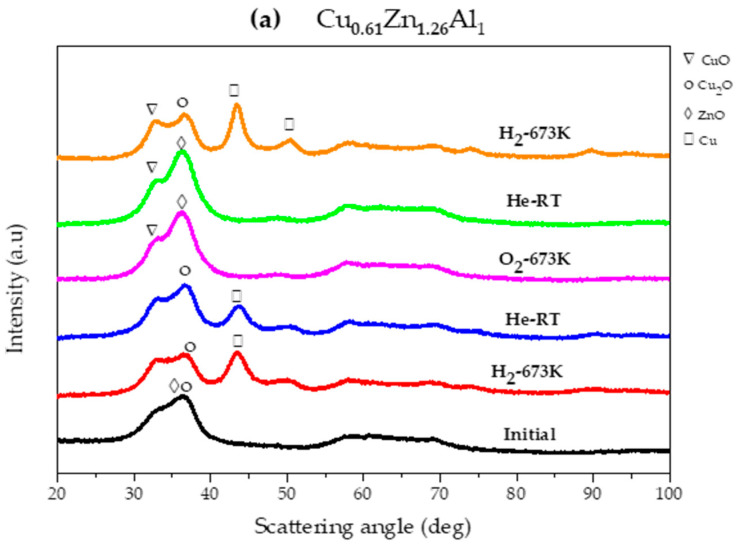

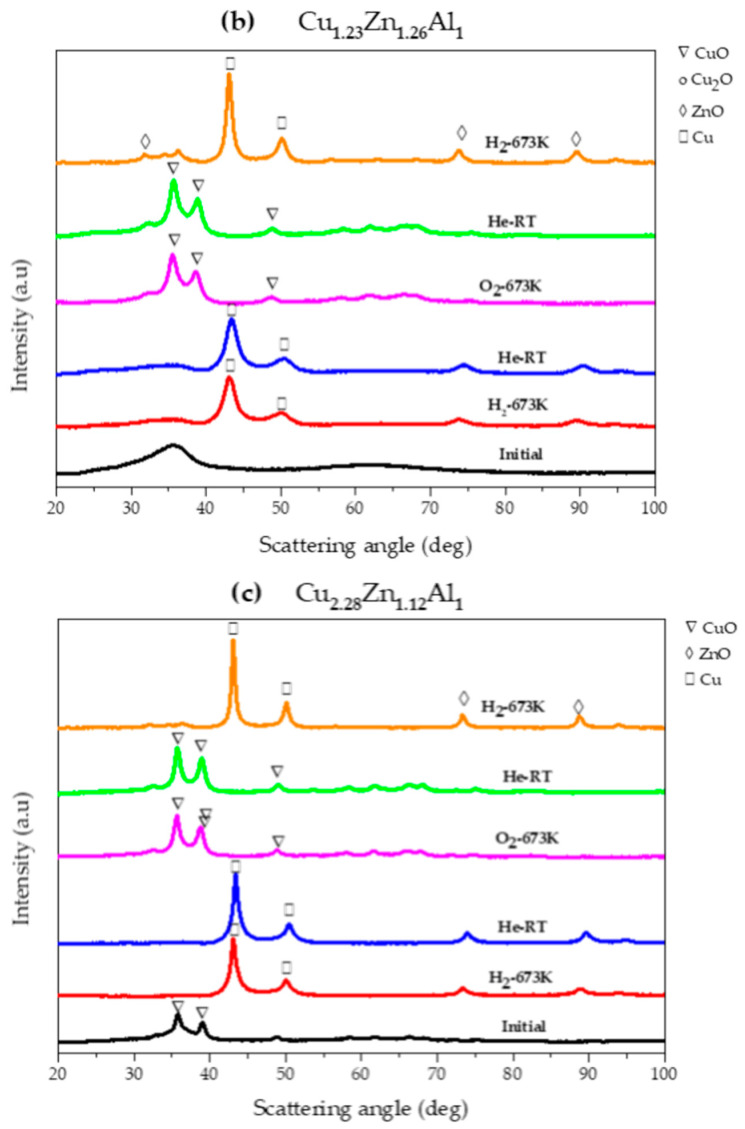
In situ XRD for (**a**) Cu**_0.61_**Zn**_1.26_**Al**_1_**, (**b**) Cu**_1.23_**Zn**_1.26_**Al**_1_**, and (**c**) Cu**_2.28_**Zn**_1.12_**Al**_1_** catalysts.

Results of the in situ XRD showed the changes that the samples undergo under the influence of treatment. Figure 2 contains diffractograms for the samples (a) Cu**_0.61_**Zn**_1.26_**Al**_1_**, (b) Cu**_1.23_**Zn**_1.26_**Al**_1_**, and (c) Cu**_2.28_**Zn**_1.12_**Al**_1_** on different stages of their biography. Initial measurements in He and O_2_ flow, at room temperature (RT), for the samples preliminary calcined outside the apparatus, revealed that all three samples differ markedly and the initial calcination led to a different state of decomposition of the original hydrotalcite structure. The recorded patterns may suggest some contribution of the Cu_2_O phase in the Cu**_0.61_**Zn**_1.26_**Al**_1_** sample and the evident presence of CuO Tenorite in the Cu**_2.28_**Zn**_1.12_**Al**_1_** sample. In addition, the diffractogram for the initial sample of Cu**_1.23_**Zn**_1.26_**Al**_1_** revealed the amorphous structure with two dominant bonds at 2θ = 30–40° and 50–80°. Moreover, for Cu**_0.61_**Zn**_1.26_**Al**_1_**, broad intensity bands coinciding roughly with a range of intense peaks of ZnO (Zincite, Wurtzite structure COD #96-900-8878) were detected. Additionally, there is a potential contribution from a highly strained (semi-amorphous) hydrotalcite polytype structure (e.g., 3R1, COD #96-300-0049), or the delafossite group, known for its intricate oxidation/reduction behavior. 

Reduction of Cu**_0.61_**Zn**_1.26_**Al**_1_** in H_2_ at 673 K causes the appearance of Cu nanoparticles (crystal size ~5 nm) at 2θ value of 43.42° representing (111) plane of fcc structure of copper and also the presence of Cu_2_O phase. Cooling this sample in He led to decomposing and decreasing Cu(111) intensity instead of the expected rise. Moreover, the heating with O_2_ at 673 K raises a broad band at 2θ = 30–40° attributed to Zincite and the oxidation of Cu (Cu_2_O or CuO). Subsequent heating in H_2_ at 673 K again leads to the formation of Cu in metallic form with a slightly larger crystallite size of ~5 nm (Figure 2a). 

Regarding the sample Cu**_1.23_**Zn**_1.26_**Al**_1_**, the reduction at 673 K with H_2_ presents peaks characteristic of the fcc Cu(111) at 2θ ≈ 43.15° with 5 nm crystal size. The visible “bridge” between (111) and (200) peaks strongly suggests its multi-twinned morphology (prone to 111 stacking faults) [26]. A further flush with He at RT, exposition to oxygen at 673 K, leads to the formation of the Tenorite CuO phase (COD #96-901-6327, Tenorite, monoclinic space group C1c1, a = 4.693 Å, b = 3.428 Å, c = 5.137 Å, β = 99.546°). A final reduction in H_2_ at 673 K displays again the fcc Cu phase (crystal size ~10 nm) together with peaks of ZnO (PDF #00-089-1397 ZnO, Wurtzite structure of hexagonal crystal system (a = 3.253 Å, c = 5.213 Å, space group P6_3_mc) (Figure 2b).

Regarding the sample Cu**_2.28_**Zn**_1.12_**Al**_1_**, the reduction in hydrogen at 673 K causes a marked decrease in the amorphous component, with the appearance of metallic Cu(111) (2θ = 43.15°) with a large crystal size (~10 nm) compared to the other samples. Heating the sample in oxygen at 673 K recovers Tenorite CuO peaks, with the phase growing in intensity and improving the long-range order as compared to the initial pattern. Only after the next round of heating in hydrogen, together with sharper Cu peaks (crystal size ~16 nm), peaks of crystalline ZnO Wurtzite structure (Figure 2c) appear. 

The cooling down to RT with He leads to the formation of Cu^+^ (Cu_2_O phase) for the three catalysts. The presence of this phenomenon could be related to the migration of oxygen from the hydrotalcite structure to the surface, which leads to the oxidation of copper. To summarize, different phases are present, after the reduction, on the surface of CuZnAl systems, which could be responsible for the difference in their catalytic performance. 

### 2.4. Transmission Electron Microscopy (TEM)

**Figure 3:**
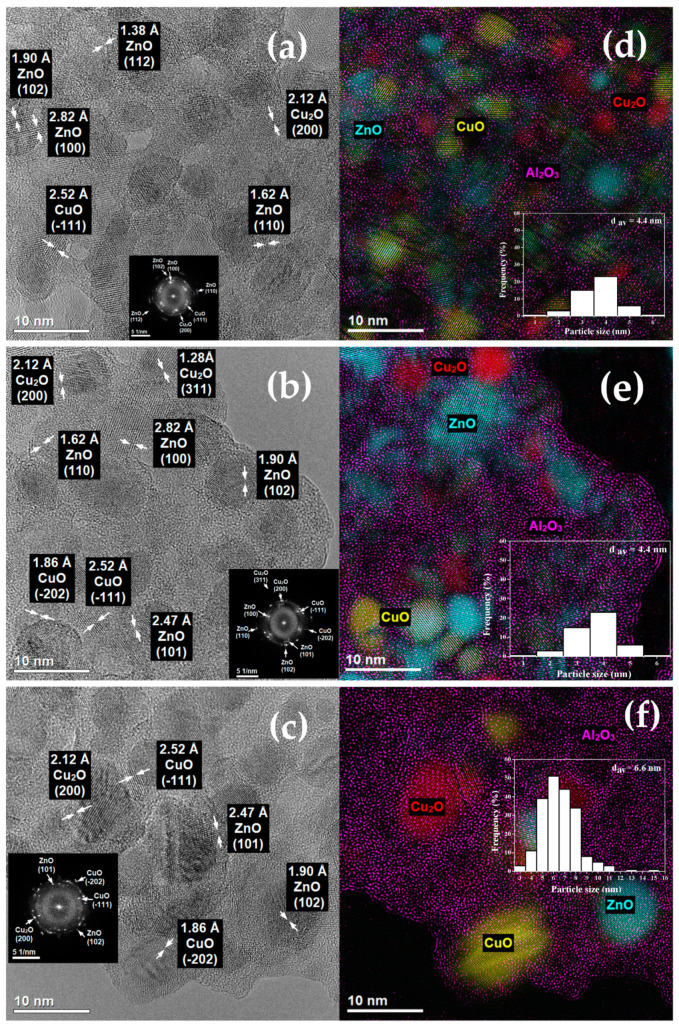
Transmission electron microscopy (TEM) images of (**a**,**d**) Cu**_0.61_**Zn**_1.26_**Al**_1_**, (**b**,**e**) Cu**_1.23_**Zn**_1.26_**Al**_1_**, and (**c**,**f**) Cu**_2.28_**Zn**_1.12_**Al**_1_**.

The HRTEM and STEM-EDS and metal particle size distribution results are presented in Figure 3a,d Cu**_0.61_**Zn**_1.26_**Al**_1_**, Figure 3b,e Cu**_1.23_**Zn**_1.26_**Al**_1_**, and Figure 3c,f Cu**_2.28_**Zn**_1.12_**Al**_1_**. The HRTEM images (Figure 3a–c) and elemental mapping (Figure 3d–f) revealed the presence of copper oxides (CuO and Cu_2_O), zinc oxide (ZnO), and aluminum oxide (Al_2_O_3_) for the three catalysts. Moreover, the particle size slightly increases with the increase in copper loading. The average particle sizes are 4.4, 4.6, and 6.6 nm for Cu**_0.61_**Zn**_1.26_**Al**_1_**, Cu**_1.23_**Zn**_1.26_**Al**_1__,_** and Cu**_2.28_**Zn**_1.12_**Al**_1_**, respectively. There is no difference between the morphology or particle size between the three CuZnAl catalysts.

### 2.5. X-ray Photoelectron Spectroscopy (XPS)

XPS studies were investigated precisely for the same area of the samples, after calcination and directly after activation, to observe the transformation of the surface during in situ activation processes. The identification of the chemical state of the metals Cu, Zn, and Al was carried out based on the photoelectron spectra of Cu 3p, Cu 3s, Cu 2p, Zn 3d, Zn 2p_3/2_, Al 2p, and Al 2s of the valence band. The chemical state of oxygen and carbon was based on the O 1s and C 1s photoelectron signals. 

The chemical state analysis of copper for three CuZnAl systems showed the presence of Cu^+^ and Cu^0^ (Cu_2_O) (BE = 932 eV) (Figure S1). Additionally, Cu**_0.61_**Zn**_1.26_**Al**_1_** and Cu**_1.23_**Zn**_1.26_**Al**_1_** also show Cu^2+^ (CuO) peaks (BE = 933 eV) and typical broad Cu^2+^ satellites. However, as a result of the reduction of Cu**_2.28_**Zn**_1.12_**Al**_1_**, the signals corresponding to Cu^2+^ (CuO, Cu(OH)_2_) were transformed entirely into Cu^+^ (Cu_2_O) (Figure S1). The analysis of the Auger spectra of Cu LMM XAES for Cu**_0.61_**Zn**_1.26_**Al**_1_** and Cu**_1.23_**Zn**_1.26_**Al**_1_** catalysts has been discussed previously [27]. For Cu**_2.28_**Zn**_1.12_**Al**_1_**, the Auger spectra reveal a kinetic energy peak at 918.8 eV, indicating the presence of metallic Cu^0^. Additionally, the presence of a second peak at 917.2 eV indicated the existence of Cu^+^ [28].

Analogically, surface analysis for zinc showed the presence of surface zinc in the form of Zn^2+^ ions in the form of ZnO, Zn(OH)_2,_ and/or ZnAl_2_O_4_ (Figure S2). High-resolution spectra of Zn 2p_3/2_ showed a single peak at 1021 eV, which indicated the presence of Zn^2+^ (ZnO, Zn(OH)_2_). The position of this peak is slightly shifted towards higher energies compared to the values reported in the literature [29], which may be due to a different chemical environment attributed to the presence of the Zn-O-Cu bond. The higher electronegativity of Cu compared to Zn should cause a shift of electron density towards copper in the Zn-O-Cu configuration, increasing the charge deficit in Zn atoms [30]. Moreover, for Cu**_2.28_**Zn**_1.12_**Al**_1_**, a Zn 3d peak at 10.6 eV was also observed, confirming the presence of Zn^2+^ (ZnO).

The XPS results for CuZnAl also reveal the presence of Al 2p and Al 2s signals at BE = 74.1 and 119 eV, respectively, which are related to the surface presence of Al (Figure S3). For CuZnAl catalysts, aluminum existed in various Al^3+^ forms: Al_2_O_3_, Al(OH)_3_, AlOOH, and/or ZnAl_2_O_4_. Despite the described results, the Al analysis was only estimated because the Al-related signals occurred in the company of Cu 3p and Cu 3s signals, which required deconvolution with some error. Nevertheless, this analysis allowed us to determine the general trends of CuZnAl systems, the impact of the reduction process on the surface, and the amount of copper on the surface of the catalysts.

Samples after reduction with hydrogen still showed O 1s signals (BE = 531 eV), which may be related to the oxidation state of Cu (Cu^0^, Cu^+^, Cu^2+^), Al (Al_2_O_3_, Al(OH)_3_), and Zn (ZnO) and the presence of CO_3_^2−^ anions on the surface (Figure S4). Activation of the CuZnAl systems caused a decrease in the surface oxygen concentration, which is an expected phenomenon related to the reaction of surface oxygen with hydrogen.

Analysis of the surface in terms of the presence of C, in the form of C 1s peaks, showed a residual amount of carbon in the form of CO_3_^2−^ ions (BE = 290 eV), which is consistent with the results obtained for oxygen and C-C/C-H bonds (BE = 285 eV), which probably come from metal precursors (Figure S4).

The comparison of the results obtained for the three samples after calcination and activation is presented in Table S1. A negligible Al, Zn, and Cu content increase after activation is observed for Cu**_0.61_**Zn**_1.26_**Al**_1_**. In the case of the reduced Cu**_1.23_**Zn**_1.26_**Al**_1_** sample, the surface was enriched with Cu and Zn and depleted of Al. On the other hand, the Cu and Zn content decreased and the Al increased on the surface of Cu**_2.28_**Zn**_1.12_**Al**_1_** compared to the calcined sample. In summary, the differences between the surface chemical compositions of CuZnAl samples may be related to differences in their catalytic efficiency.

### 2.6. NH_3_ and CO_2_ Temperature-Programmed Desorption (NH_3_-TPD and CO_2_-TPD)

The NH_3_-TPD and CO_2_-TPD profiles of CuZnAl catalysts are shown in Figure S5a,b, respectively. The calculated surface acidic sites and basic site concentrations are presented in Table S2 and Table S3, respectively. The results showed that increasing Cu loading led to enhanced surface acidity. All the catalysts showed the presence of weak, moderate, and strong acid sites confirmed by the NH_3_ desorption maxima at 323–473 K, 473–673 K, and above 673 K [31], respectively (Figure S5a and Table S2). 

On the other hand, the CO_2_-TPD results revealed the existence of both Lewis and Brønsted basic sites on different strengths on the surface of the catalysts. The deconvolution of the profiles into three Gaussian/Lorenzian peaks assigned them to weak (α), moderate (β), and strong (γ) basic sites [27] (Figure S5b and Table S3).

### 2.7. Catalytic Test

**Figure 4:**
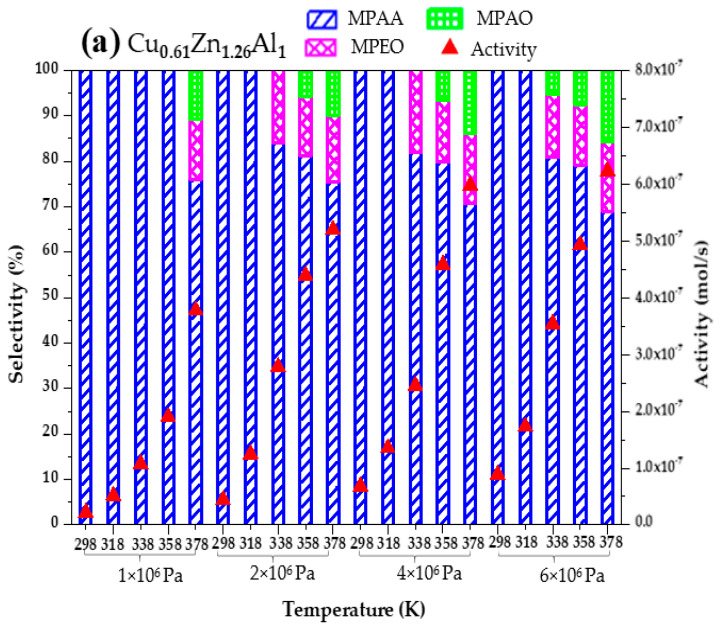

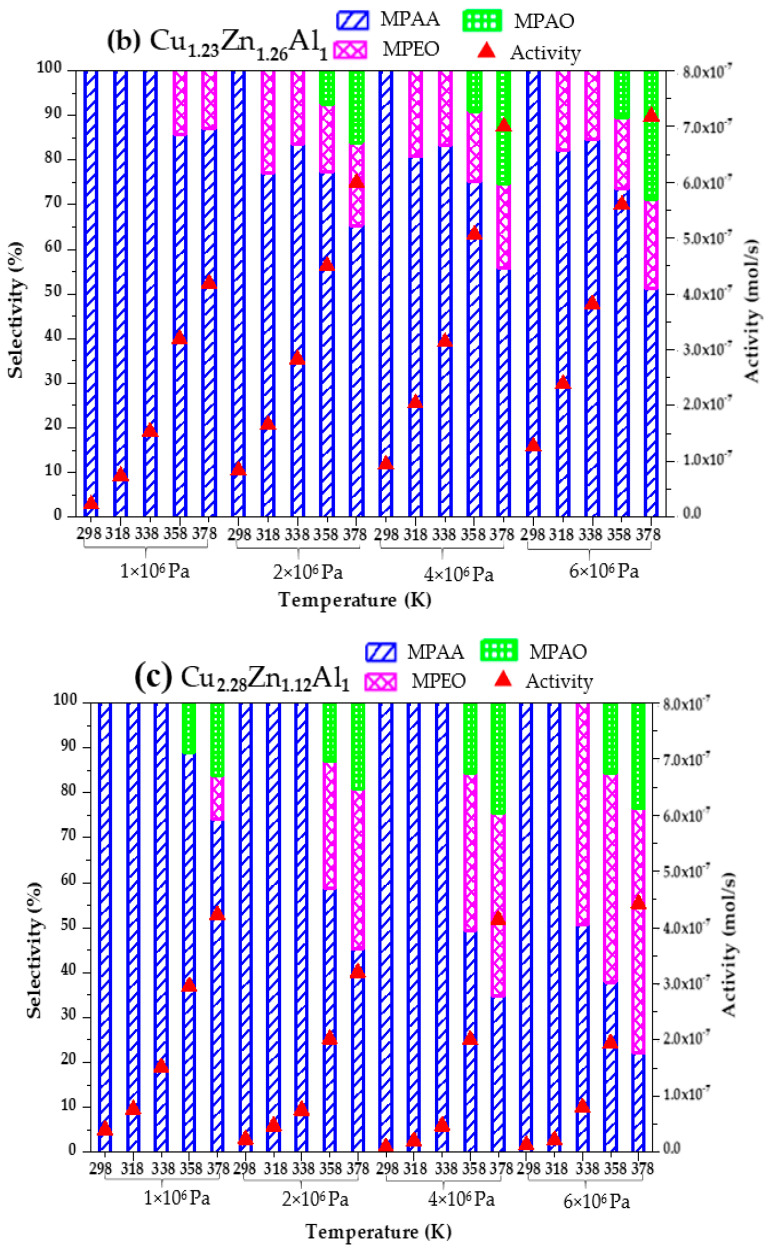
Catalytic results for (**a**) Cu**_0.61_**Zn**_1.26_**Al**_1_**, (**b**) Cu**_1.23_**Zn**_1.26_**Al**_1_**, and (**c**) Cu**_2.28_**Zn**_1.12_**Al**_1_** catalysts.

The catalytic activity of three CuZnAl systems was examined in the continuous-flow chemoselective hydrogenation of 2-methyl-2-pentenal. Each time, 0.100 g of freshly reduced catalyst was used for the reaction. The influence of pressure and temperature on this process using Cu**_0.61_**Zn**_1.26_**Al**_1_**, Cu**_1.23_**Zn**_1.26_**Al**_1_**, and Cu**_2.28_**Zn**_1.12_**Al**_1_** was also investigated. The catalytic results are shown in Figure 4: (a) for Cu**_0.61_**Zn**_1.26_**Al**_1_**, (b) for Cu**_1.23_**Zn**_1.26_**Al**_1_**, and (c) for Cu**_2.28_**Zn**_1.12_**Al**_1_**. Due to the complexity of the MPEA hydrogenation mechanism, the performance of catalysts may be affected by many factors such as the interface’s structure, composition, particle size distribution, and reaction conditions [22]. For the three catalysts, the activity increased with the increase in temperature and pressure (Figure 4a–c). The highest activity was obtained for the Cu**_1.23_**Zn**_1.26_**Al**_1_** catalyst (Figure 4b). This activity can be associated with the surface composition of this sample, and mainly with the presence of various forms of copper on the surface (Cu^0^/Cu^+^), in appropriate proportions, as was observed in the hydrogenation of crotonaldehyde [32]. However, disturbing this optimal ratio causes a decrease in substrate conversion. Based on XPS results for three CuZnAl systems (Table S1), as the Cu content in the catalyst increased, the amount of the Cu^0^ fraction decreased in favor of Cu^+^. As a consequence, the highest substrate conversion was observed for the Cu**_1.23_**Zn**_1.26_**Al**_1_** catalyst with the most favorable Cu^0^/Cu^+^ ratio, while for Cu**_0.61_**Zn**_1.26_**Al**_1_** and Cu**_2.28_**Zn**_1.12_**Al**_1_**, the amount of the Cu^+^ fraction is successively too high and too low to maximize the conversion. On the other hand, the highest activity of Cu**_1.23_**Zn**_1.26_**Al**_1_** could be related to the lowest amount of the basic sites (Table S3) in comparison to other samples [33].

In summary, the results revealed that the surface catalyst’s chemical environment affects the activity of CuZnAl hydrotalcite-derived materials in the chemoselective hydrogenation of MPEA. 

Moreover, catalytic experiments have shown that temperature and pressure significantly impact the formation of the desired products: saturated aldehyde (MPAA) and unsaturated alcohol (MPEO) (Figure 4). At low temperatures (≤338 K), the hydrogenation of MPEA contributed to the formation of 100% MPAA at every pressure. However, it is usually related to low substrate conversion. The presence of, only, this compound in the reaction mixture at low reaction conditions is related to lower activation energy required for C=C bond hydrogenation than the C=O bond hydrogenation [34].

For all catalysts, with increasing pressure at higher temperatures (358–373 K), there is the formation of (MPEO) and undesired saturated alcohol (MPAO). This is especially visible for the Cu**_2.28_**Zn**_1.12_**Al**_1_** catalyst with 60% conversion and 54% selectivity to MPEO at 373 K and 6 × 10^6^ Pa (Figure 4c). 

The increase of selectivity to MPEO at high temperatures and pressures for Cu**_2.28_**Zn**_1.12_**Al**_1_** means that the reaction mechanism changes under higher temperature and pressure conditions by changing the geometry of MPEA sorption at active sites. Moreover, the increase in MPEO production for the Cu**_2.28_**Zn**_1.12_**Al**_1_** catalyst, with a decrease in conversion, despite the increase in the content of the active phase, is the dominance of the Cu(111) crystal plane confirmed by in situ XRD (Figure 2). Theoretical calculations and experimental studies showed that this is the only crystal plane on which adsorption via the C=O bond is preferred [32,34]. The absence of MPEO in the post-reaction mixture at a low-temperature range (Figure 4) can be associated with both low conversion and low energy supplied to the system, making it difficult to obtain sufficient activation energy for the adsorption of the substrate on the Cu(111) plane [32].

Moreover, the production of MPEO is related to the existence of Cu^+^ on the surface. These sites can serve as electrophilic centers, enabling the polarization of the C=O bond through interaction with the lone electron pair in oxygen. This electronic arrangement might aid in activating the electronic doublet of oxygen within the carbonyl group to a certain degree. Consequently, this mechanism could enhance the hydrogenation of the C=O group [28]. 

The spillover effect is another factor that may contribute to the production of MPEO. The presence of Zn^2+^ on the surface of the catalysts provides new active sites for aldehyde adsorption, leading to the hydrogenation of the substrate outside the copper surface. In addition, copper is active in the dissociative adsorption of H_2_ and can provide hydrogen through the spillover effect to selectively hydrogenate the C=O group on Zn^2+^ sites, leading to an increase in the production of MPEO on the catalyst surface [19]. 

Another parameter that can affect the formation of unsaturated alcohol is the delicate balance between the strength and ratio of acid–base sites present in CuZnAl hydrotalcite-derived catalysts. The highest amount of weak acid sites and moderate basic sites (Tables S2 and S3) in the Cu**_2.28_**Zn**_1.12_**Al**_1_** promote the C=O hydrogenation (Figure 4c) [35]. 

In summary, different active sites are responsible for the adsorption of MPEA via the C=O bond. This adsorption improves the hydrogenation of this group and increases the production of MPEO on the Cu**_2.28_**Zn**_1.12_**Al**_1_** catalyst surface.

The limited number of research papers addressing the hydrogenation of MPEA [5,10] makes it difficult to compare the catalytic results obtained in this reaction. However, comparing this work with the results of chemoselective hydrogenation of other α,β-unsaturated aldehydes, the 54% selectivity to MPEO with 60% substrate conversion can be described as a relatively high value, taking into account the use of readily available transition metals as catalysts. The hydrogenation of crotonaldehyde with 1.8 wt.% Cu supported on graphite carbon fibers achieved only 15.1% selectivity to unsaturated alcohol [32]. Moreover, the same work did not demonstrate the predisposition of copper catalysts to produce unsaturated aldehyde. On the other hand, the chemoselective hydrogenation of cinnamaldehyde with CuCoZnAl (0.5-0.5-1-1) led to the formation of cinnamyl alcohol with 53% selectivity and 30% substrate conversion [19]. Increasing the cinnamaldehyde conversion to 60% was accompanied by decreasing this selectivity to 36%. Compared to other transition metal catalysts, only using the CoFeB alloy with Fe: (Fe + Co) nominal molar ratio of 0.6 allowed for achieving 67% selectivity to unsaturated alcohol with 95% of the crotonaldehyde conversion [36]. However, when comparing the catalytic results using systems containing noble metals, only 1.6 wt.% was used. Au/TiO_2_ made it possible to obtain unsaturated alcohol with 51% selectivity and 21% crotonaldehyde conversion [37].

**Figure 5:**
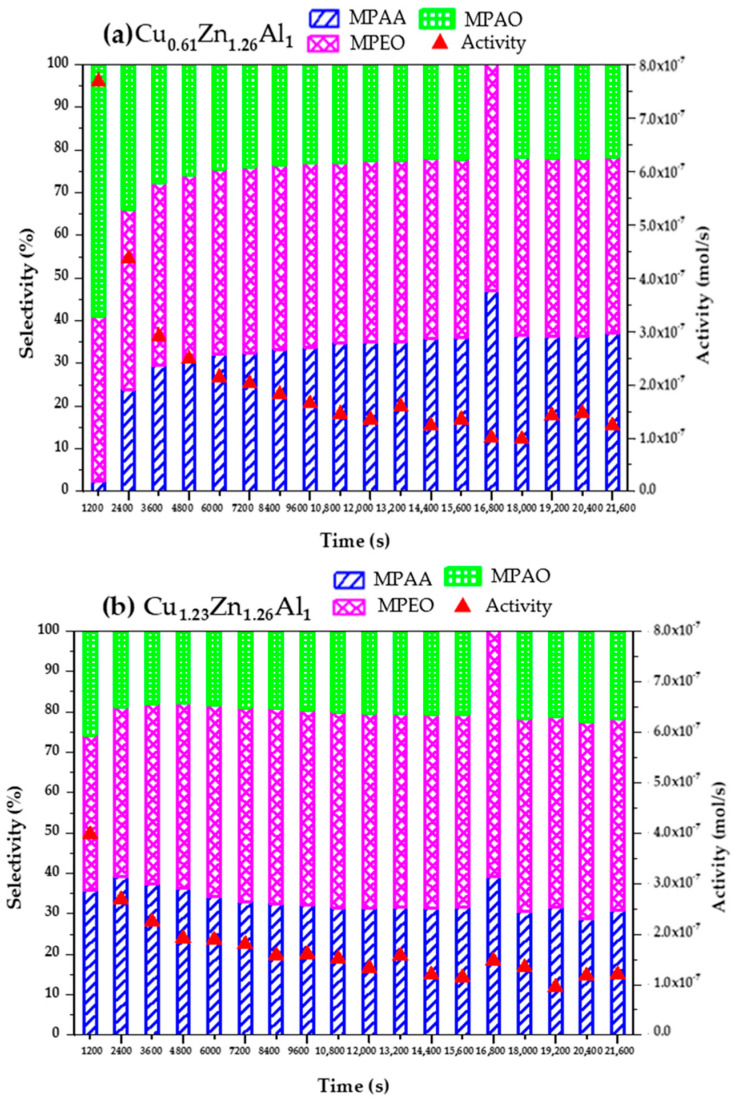

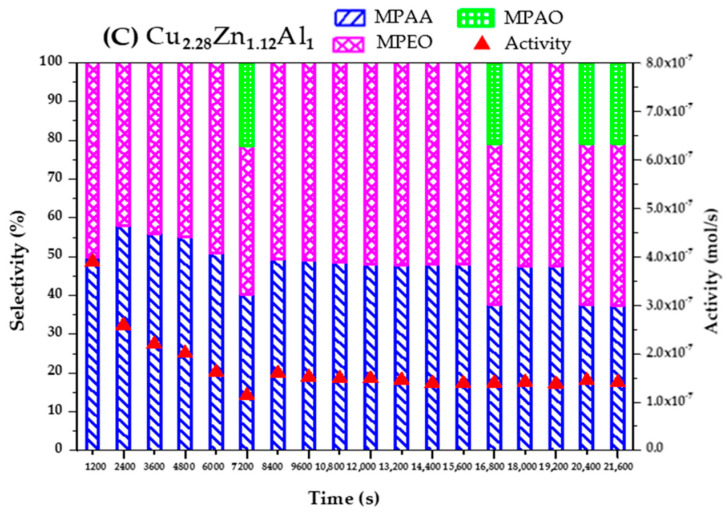
Long-term tests for (**a**) Cu**_0.61_**Zn**_1.26_**Al**_1_**, (**b**) Cu**_1.23_**Zn**_1.26_**Al**_1_**, and (**c**) Cu**_2.28_**Zn**_1.12_**Al**_1_** catalysts at 358 K and 4 × 10^6^ Pa for 6 h (21,600 s).

The screening of the catalytic performance of the CuZnAl hydrotalcite-derived materials allowed the selection of the conditions for stability tests. The long-term experiments were performed at 358 K and 4 × 10^6^ Pa for 6 h (21,600 s). The results of these tests are presented in Figure 5: (a) Cu**_0.61_**Zn**_1.26_**Al**_1_**, (b) Cu**_1.23_**Zn**_1.26_**Al**_1_**, and (c) Cu**_2.28_**Zn**_1.12_**Al**_1_**. For Cu**_0.61_**Zn**_1.26_**Al**_1_** and Cu**_1.23_**Zn**_1.26_**Al**_1_**, there is a formation of three products with different ratios throughout the test. For the catalyst Cu**_2.28_**Zn**_1.12_**Al**_1_**, there is a production of, mainly, two desired products MPAA and MPEO. In addition, the highest activity and selectivity toward desired products MPAA and MPEO was dedicated to the highest copper loadings catalyst (Figure 5c). During the initial hour of this reaction, rapid deactivation of three catalysts was observed. This observation could imply that the catalysts undergo a degree of equilibration during the initial phase of MPEA hydrogenation. On the other hand, a negligible deactivation was observed within the rest of the test. This may be due to the formation of carbon deposits on the catalyst surface during the reaction, gradually blocking the catalyst pores and covering the active sites, resulting in the deactivation of the catalysts [38,39].

In summary, the CuZnAl catalysts have two different active sites for the adsorption of 2-methyl-2-pentenal. The first sites are responsible for adsorbing MPEA molecules via the C=C bond, which achieves 100% selectivity to saturated aldehyde (MPAA) under mild conditions, revealing their potential as catalysts in the pharmaceutical industry. The second sites interact with the C=O group of MPEA molecule and produce unsaturated alcohol (MPEO), especially for the catalyst Cu**_2.28_**Zn**_1.12_**Al**_1_**. This result seems attractive because this compound’s production is challenging, expensive, and difficult to obtain. So, this catalyst plays a crucial role in increasing the availability of unsaturated alcohols, which have various applications, notably in the perfume industry [36]. 

## 3. Materials and Methods

### 3.1. Preparation of Hydrotalcite Materials

Three hydrotalcites with different Cu-Zn-Al molar ratios were synthesized by the co-precipitation method, ref. [27] in a commercial 5 L glass reactor (Syrris Globe, Royston, UK) equipped with two-piston pumps, a shaft stirrer, a pH meter, and a thermocouple. A solution of metals was prepared by dissolving the required metal nitrates (total concentration of metals not more than 1 mol/L) in distilled water. In each case, the solution of appropriate metals was introduced to the reactor simultaneously with an alkali solution prepared by the dissolution of potassium carbonate and potassium hydroxide (c(K_2_CO_3_) = 0.2 mol/L + c(KOH) = 2 mol/L) in distilled water, which maintained pH on 9.5. Co-precipitation was carried out every time at 333 K. The reaction mixture was intensively stirred and aged for 1 h after the co-precipitation process. In the next step, the solid product (layered double hydroxide; LDH) was separated from the reaction mixture using a filter press and washed with deionized water to remove the sodium ions from the catalysts. After synthesis, hydrotalcite materials were calcined in air for 3 h at 773 K (ramp 0.05 K/s), forming mixed oxides. The activation step for each material was performed at 673 K for 3 h (ramp 0.2 K/s) in a stream of 10% H_2_/Ar. 

### 3.2. Materials Characterization

The samples were analyzed with different techniques to determine their physicochemical properties; inductively coupled plasma-optical emission spectroscopy (ICP-OES), temperature-programmed reduction (TPR), in situ X-ray diffraction (in situ XRD) [40], transmission electron microscopy (TEM) measurement, X-ray photoelectron spectroscopy (XPS) [41,42,43], and NH_3_ and CO_2_ temperature-programmed desorptions (NH_3_-TPD and CO_2_-TPD). A detailed description of these methods is provided in Supplementary Materials. 

### 3.3. Catalytic Tests

The continuous-flow chemoselective hydrogenation of 1 wt.% 2-methyl-2-pentenal (MPEA) in ethanol was conducted in the ThalesNano H-Cube device (Budapest, Hungary), described in detail in the previous work [27]. Each experiment used 100 mg of the catalyst, a 0.5 mL of solution per minute flow rate, and an H_2_ flow rate of 1 mL/s. The residence time (defined as bed volume/flow rate) was 1.6 min. Different pressure conditions (1 × 10^6^, 2 × 10^6^, 4 × 10^6^, and 6 × 10^6^ Pa) and temperatures (298 K, 318 K, 338 K, 358, and 373 K) were investigated. The obtained samples were analyzed in a gas chromatographic system (Bruker 456-GC with FID detector) equipped with a BP5 column (30 m, 0.25 mm, and 0.25 m).

In addition, the stability tests of the three catalysts were carried out under isothermal and isobaric conditions (358 K and 4 × 10^6^ Pa) for 6 h (21,600 s). These long-term experiments were performed at the same initial concentration of MPEA and flow conditions as those employed during screening. 

## 4. Conclusions

Copper oxidation states and surface properties significantly influence the liquid-phase continuous-flow chemoselective hydrogenation of 2-methyl-2-pentenal on CuZnAl hydrotalcite-derived materials. Furthermore, it elucidates the pivotal role that surface-bound Cu species play in dictating reaction pathways and selectivity. All catalysts produce MPAA (desired product) with 100% selectivity at low temperatures and pressures. Moreover, the catalytic activity decreased with increasing Cu loading, which is related to the Cu^0^/Cu^+^ ratio. At high temperatures and pressures, the production of MPEO increased especially for the catalyst Cu**_2.28_**Zn**_1.12_**Al**_1_** with 54% selectivity and 60% conversion at 378 K and 6 × 10^6^ Pa. This phenomenon can be attributed to the presence of active sites on the catalyst surface that facilitate the adsorption of the reactant molecule through the C=O bond. This interaction with C=O adsorption sites leads to an enhanced tendency for the formation of MPEO. The delicate balance between these adsorption pathways contributes to the observed selectivity alteration. This observation underscores the complex interplay among surface properties, adsorption mechanisms, and reaction conditions, ultimately shaping the distribution of products in catalytic processes.

## Data Availability

The original contributions presented in the study are included in the article; further inquiries can be directed to the corresponding authors.

## Supplementary Materials

The following supporting information can be downloaded at https://www.mdpi.com/article/10.3390/molecules29143345/s1, Detailed description of the methods, Figure S1: XPS results—Cu 2p for Cu**_0.61_**Zn**_1.26_**Al**_1_**, Cu**_1.23_**Zn**_1.26_**Al**_1_**, and Cu**_2.28_**Zn**_1.12_**Al**_1_**, Figure S2: XPS results—Zn 2p for Cu**_0.61_**Zn**_1.26_**Al**_1_**, Cu**_1.23_**Zn**_1.26_**Al**_1_**, and Cu**_2.28_**Zn**_1.12_**Al**_1_**, Figure S3: XPS results—Al 2p and Al 2s for Cu**_0.61_**Zn**_1.26_**Al**_1_**, Cu**_1.23_**Zn**_1.26_**Al**_1_**, and Cu**_2.28_**Zn**_1.12_**Al**_1_**, Figure S4: XPS results—O 1s and C 1s for Cu**_0.61_**Zn**_1.26_**Al**_1_**, Cu**_1.23_**Zn**_1.26_**Al**_1_**, and Cu**_2.28_**Zn**_1.12_**Al**_1_** of the studied three samples at the initial state, Figure S5: (a) NH_3_-TPD and (b) CO_2_-TPD profiles of Cu**_0.61_**Zn**_1.26_**Al**_1_**, Cu**_1.23_**Zn**_1.26_**Al**_1_**, and Cu**_2.28_**Zn**_1.12_**Al**_1_** catalysts, Table S1: XPS results for CuZnAl catalysts after calcination and in situ activation, Table S2: Distribution of acidic sites on reduced CuZnAl catalysts, Table S3: Distribution of basic sites on CuZnAl catalysts.
