# Supplementary material for: Catalytic Performance of CuZnAl Hydrotalcite-Derived Materials in the Continuous-Flow Chemoselective Hydrogenation of 2-Methyl-2-pentanal toward Fine Chemicals and Pharmaceutical Intermediates
Source: Molecules. 2024 Jul 16;29(14):3345. doi: 10.3390/molecules29143345 (PMC11279871; doi:10.3390/molecules29143345)
Supplement: Supplementary file 1 [file molecules-29-03345-s001.zip › molecules-3072644-supplementary.pdf]

Supplementary material

Catalytic Performance of CuZnAl Hydrotalcite-Derived Materials in the Continuous-Flow Chemoselective Hydrogenation of 2-Methyl-2-pentanal toward Fine Chemicals and Pharmaceutical Intermediates

Rahma Abid [1,*], Bartosz Zawadzki [1], Jaroslav Kocik [2], Grzegorz Słowik [3], Janusz Ryczkowski [3], Mirosław Krawczyk [1], Zbigniew Kaszkur [1], Izabela S. Pieta [1] and Anna Śrębowata [1*]

[1] Institute of Physical Chemistry, Polish Academy of Sciences, ul. Kasprzaka 44/52 PL, 01-224, Warsaw, Poland; rabid@ichf.edu.pl (R.A.); bzawadzki394@gmail.com (B.Z); mkrawczyk@ichf.edu.pl (M.K); zkaszkur@ichf.edu.pl (Z.K); ipieta@ichf.edu.pl (I.S.P); asrebowata@ichf.edu.pl (A.S).

[2] ORLEN UniCRE a.s, Záluží 1, 436 70 Litvínov; Czech Republic; jaroslav.kocik@orlenunicre.cz (J.K)

[3] Department of Chemical Technology, Faculty of Chemistry, Maria Curie- Skłodowska University, Plac Maria Curie-Skłodowskiej 3, 20-031, Lublin Poland; grzesiek.slowik@gmail.com (G.S); janusz.ryczkowski@mail.umcs.pl (J.R)

Materials and Methods

1. Inductively Coupled Plasma - Optical Emission Spectroscopy (ICP-OES)

ICP-OES measurements were investigated with the ICP-OES Agilent 725 device. Before every measurement, the solution containing the CuZnAl hydrotalcite-derived materials dissolved in H_2SO_4 solution (1:1) was heated to 373 K, furthermore cooled down to 298 K and diluted with demineralized water and again heated to 373 K and kept at this temperature for 600 s. Then, the solution was transferred into a volumetric flask and measurements were taken for every sample.

2**.** *Temperature-programmed reduction (TPR)*

Temperature-programmed reduction (TPR) of CuZnAl mixed oxides was conducted in the homemade glass gas flow system equipped with a thermal conductivity detector (TCD). Each time, 100 mg of the sample was heated in a quartz reactor to 1100 K (ramp 0.2 K/s) in the 10 % H_2/Ar stream. Injections of known amounts of hydrogen into the hydrogen–argon flow were provided for calibration (before and after each TPR run).

3. In Situ X-ray Diffraction

The measurements were done using the diffractometric in situ setup described in the work of Zieliński, M.et al [40]. The diffractometer (Bruker AXS D5000) operated in Bragg-Brentano geometry and used a LynxEye strip detector. The Cu-sealed tube was charged with 40 kV and 40 mA. Data were

collected for 2θ ranging from 10° to 100°, 0.02°/sec, lasting about 4500s. The initial measurements, for the calcined materials, were performed in air and Helium (He). Afterward, the samples were heated from room temperature to 673K in He/H_2 (0.7/0.03 ml/s) with a heating rate of 0.2 K/s, then cooled down to room temperature (RT) in He. Later, the samples were exposed to O_2 (4ml) and He (20ml) at 673 K and cooled down to RT in He. Next, the materials were reduced again with H_2 at 673K. The crystallite sizes of metallic Cu were calculated by the Scherrer equation.

4. Transmission Electron Microscopy (TEM)

Transmission electron microscopy (TEM) investigations were conducted using a JEOL JEM-100 CXII electron microscope from Tokyo, Japan, and using Titan G2 60- 300 kV (FEI Company) equipped with the field emission gun (FEG), monochromator, three condenser lenses system, the objective lens system, image correction (Cs-corrector), HAADF detector and EDS (Energy Dispersive X-Ray Spectroscopy) spectrometer. The microscopes were operated at an acceleration voltage of 100 keV and 300 keV, respectively. To prepare the samples for TEM analysis, they were first dispersed in pure alcohol using an ultrasonic cleaner. A drop of this suspension was then placed onto carbon films on copper grids. At least 300 particles were analyzed to create size distribution histograms. The element mapping was carried out in the STEM mode by collecting point-by-point EDS spectrum for each of the corresponding pixels in the map. The collected maps were presented in the form of a matrix of colored pixels with intensity corresponding to the amount of the given element in the mapped place of the catalyst sample.

5. X-ray photoelectron spectroscopy (XPS)

XPS measurements are widely used for the characterization of the chemical composition and oxidation states of materials. In this case, the PHI 5000 VersaProbe spectrometer with monochromatic Al Kα radiation was used to perform XPS measurements on the CuZnAl catalysts. The hemispherical analyzer was set at a pass energy of 23.5 with an energy step size of 0.1 eV to collect high-resolution XPS spectra. The X-ray beam was incident at a 45° angle to the sample surface, and the analyser axis was located at the same angle. Casa XPS software was used to analyse the XPS data, which involved deconvoluting the spectra using a Shirley background and a Gaussian peak shape with 30% Lorentzian character. The chemical states of Cu, Zn, Al, O, and C were identified based on photoelectron and Auger electron signals from Cu 2p, Cu LMM, Zn 2p3/2, Al 2p, Al 2s, O1s, and C 1s, using literature data and an electronic XPS database [41-43].

6. NH_3 and CO_2 temperature-programmed desorption (NH_3-TPD and CO_2-TPD)

NH_3 and CO_2 temperature-programmed desorptions (NH_3-TPD and CO_2-TPD) were applied to measure the acid-base properties of the catalyst samples. The runs were performed in a quartz tube reactor connected to a quadrupole mass spectrometer (HPR 60, Hiden). 50 mg of the catalyst sample

was used for each test. Prior to each measurement, the pre-reduced sample was treated with helium at 773K for 1 h to remove the adsorbed impurities. After cooling to 323K under a He flow, the sample was exposed to 50 ml·min-1 flow of 5% NH_3-He mixture or 5% CO_2-He (in the case of NH_3-TPD or CO_2-TPD experiment, respectively) for 1800 s, followed by purging with He for 1800 s to remove all physically adsorbed molecules. Afterward, the TPD experiment was started up to 773K with a heating rate of 0.2 K/s under He flow (0.8 ml/s). NH_3 and CO_2 peaks areas were quantitatively calibrated by injecting NH_3 and CO_2 pulses.

X-ray photoelectron spectroscopy (XPS)

Cu 2p

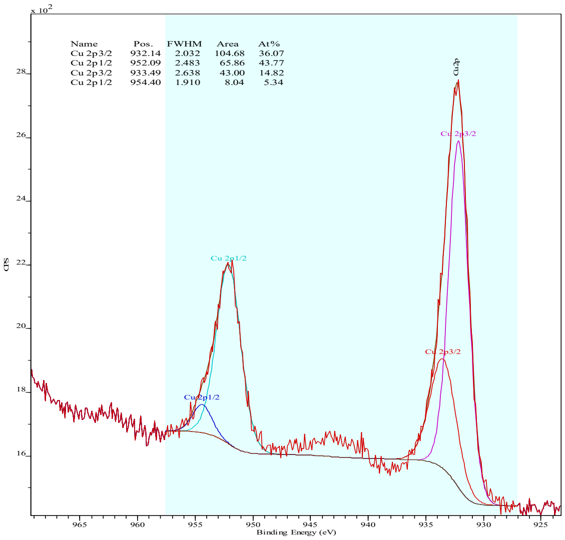

$Cu_{0.61}$ $Zn_{1.26}$ Al_1

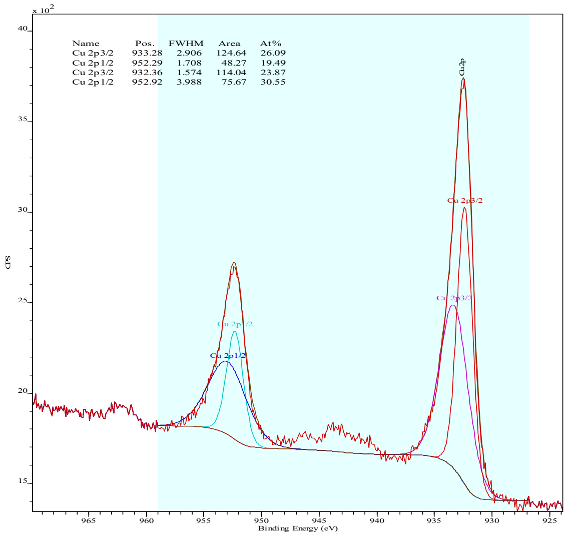

$Cu_{1.23}$ $Zn_{1.26}$ Al_1

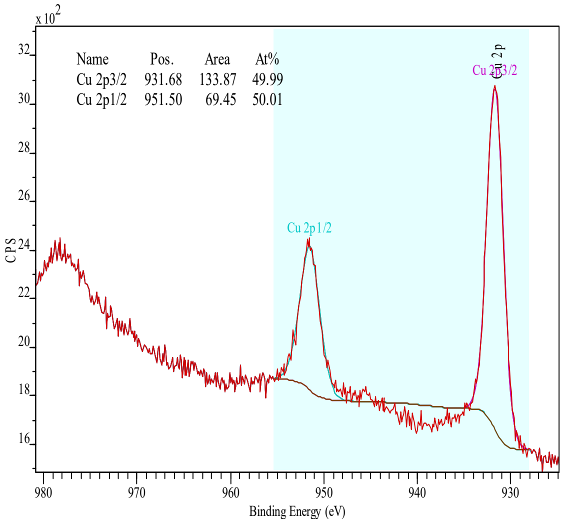

$Cu_{2.28}$ $Zn_{1.12}$ Al_1

Figure S1. XPS results - Cu 2p for $Cu_{0.61}$ $Zn_{1.26}$ Al_1, $Cu_{1.23}$ $Zn_{1.26}$ Al_1 and $Cu_{2.28}$ $Zn_{1.12}$ Al_1.

Zn 2p

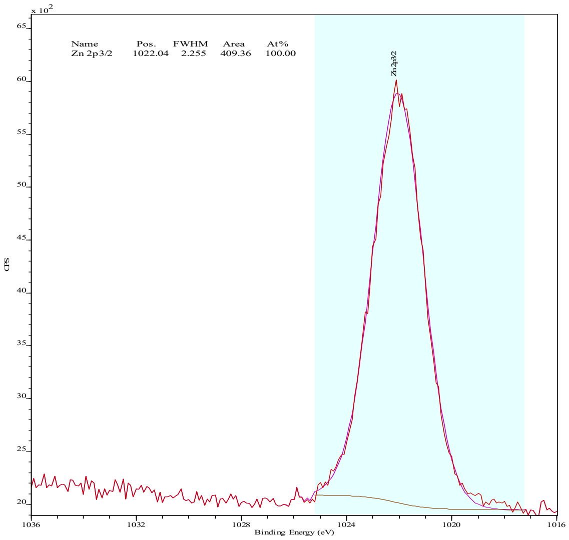

$Cu_{0.61}$ $Zn_{1.26}$ Al_1

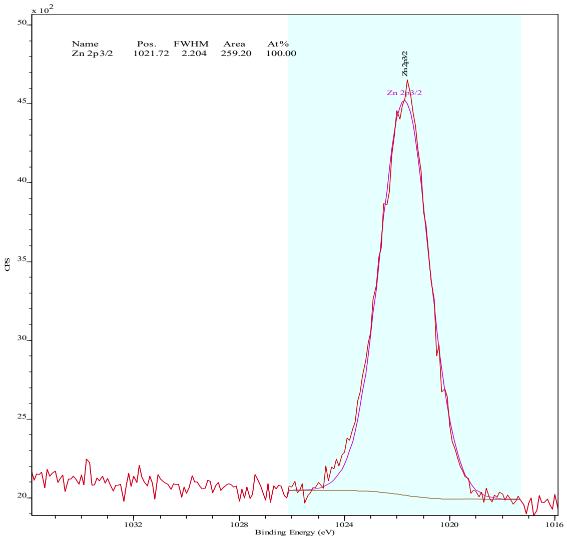

$Cu_{1.23}$ $Zn_{1.26}$ Al_1

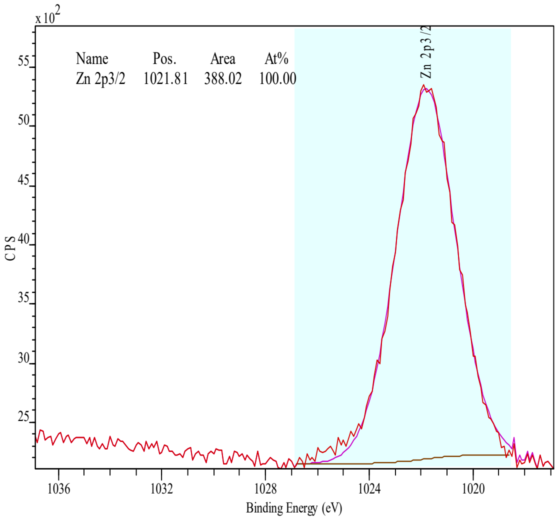

$Cu_{2.28}$ $Zn_{1.12}$ Al_1

Figure S2. XPS results - Zn 2p for $Cu_{0.61}$ $Zn_{1.26}$ Al_1, $Cu_{1.23}$ $Zn_{1.26}$ Al_1 and $Cu_{2.28}$ $Zn_{1.12}$ Al_1.

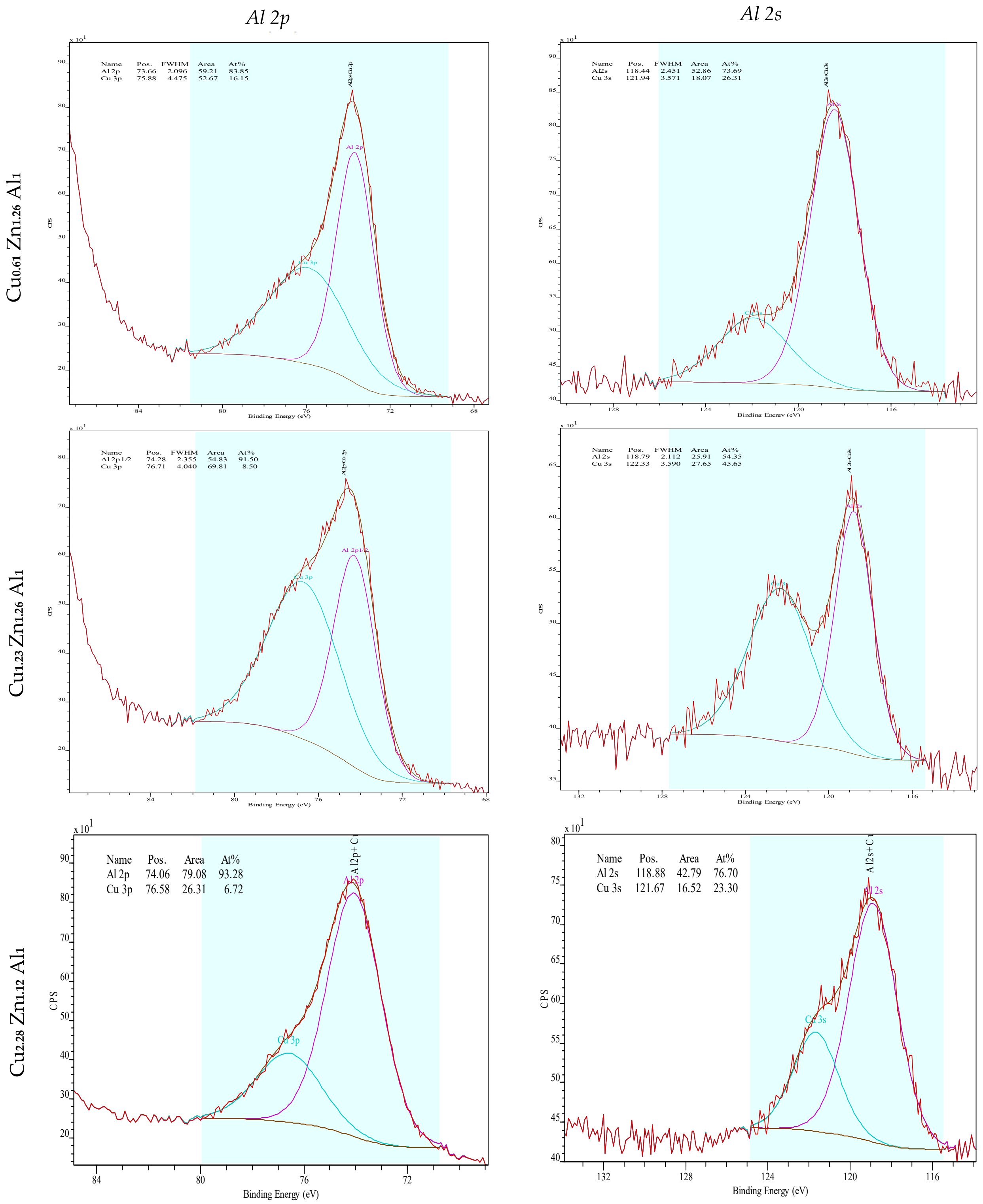

Figure S3. XPS results - Al 2p and Al 2s for $Cu_{0.61}$ $Zn_{1.26}$ Al_1, $Cu_{1.23}$ $Zn_{1.26}$ Al_1 and $Cu_{2.28}$ $Zn_{1.12}$ Al_1.

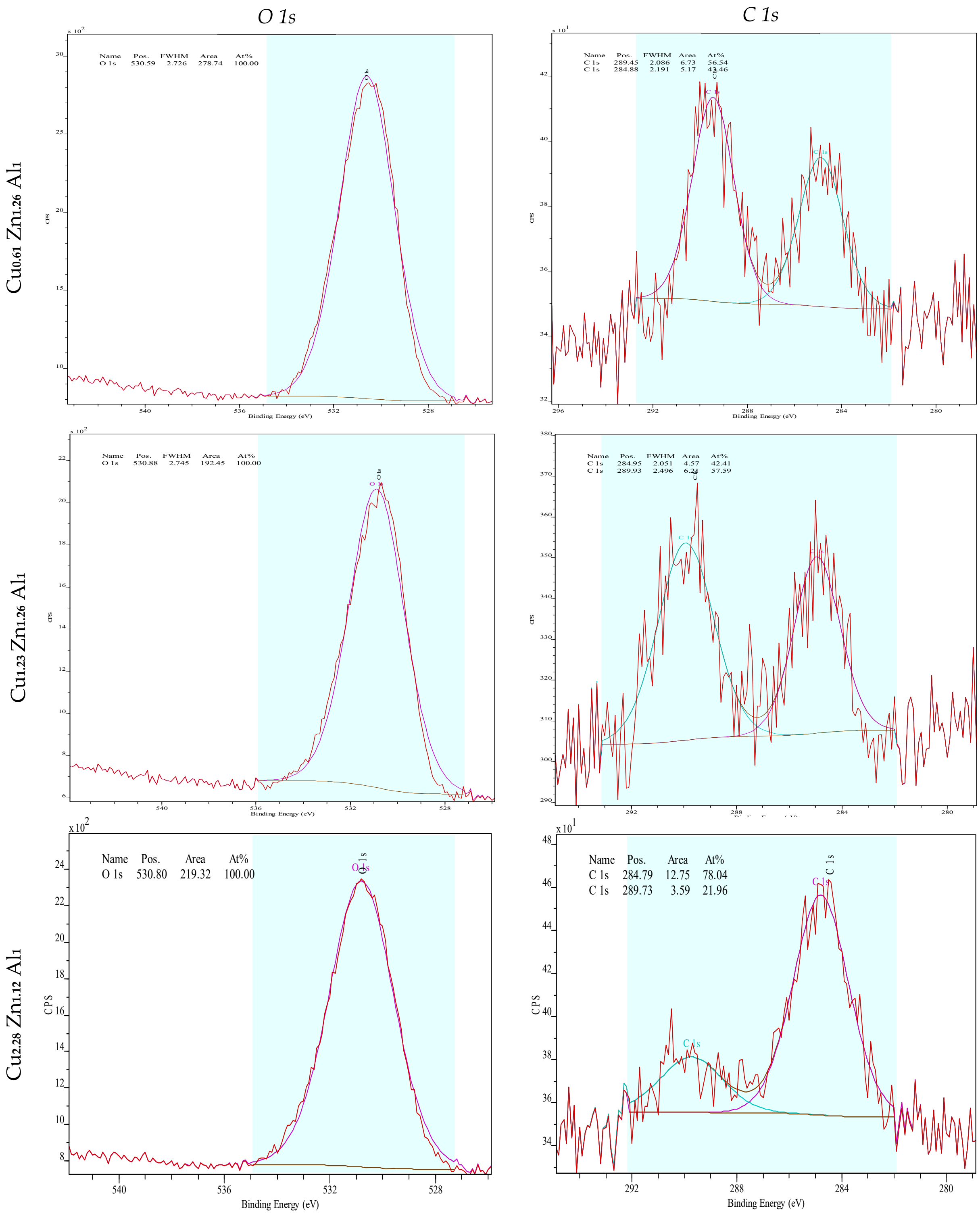

Figure S4. XPS results - O 1s and C 1s for $Cu_{0.61}$ $Zn_{1.26}$ Al_1, $Cu_{1.23}$ $Zn_{1.26}$ Al_1 and $Cu_{2.28}$ $Zn_{1.12}$ Al_1 of the studied three samples at the initial state.

NH_3 and CO_2 temperature-programmed desorption

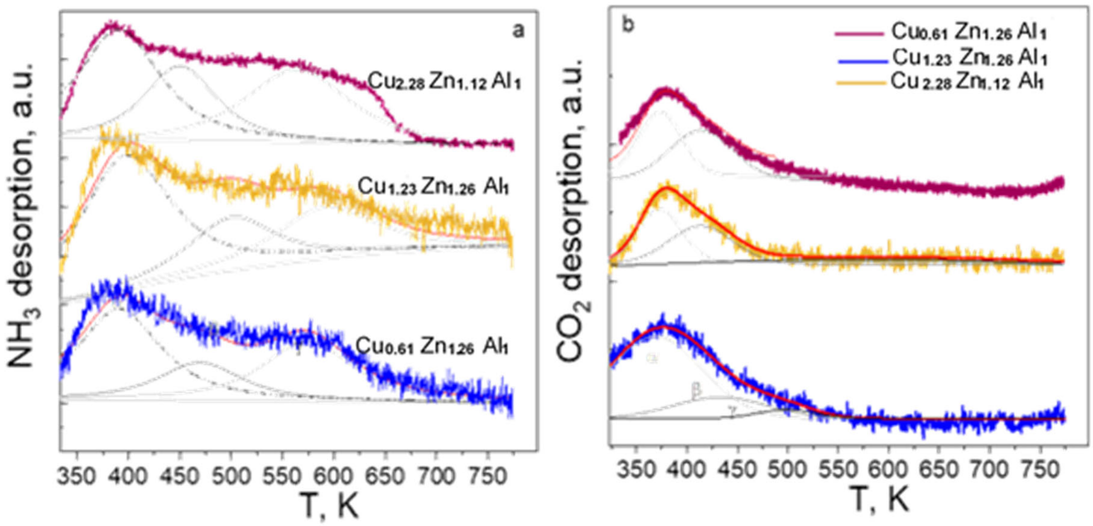

Figure S5. (a) NH_3-TPD and (b) CO_2-TPD profiles of $Cu_{0.61}Zn_{1.26}Al_1$, $Cu_{1.23}Zn_{1.26}Al_1$ and $Cu_{2.28}Zn_{1.12}Al_1$ catalysts.

Table S1: XPS results for CuZnAl catalysts after calcination and in situ activation.

	$Cu_{0.61}Zn_{1.26}Al_1$		$Cu_{1.23}Zn_{1.26}Al_1$		$Cu_{2.28}Zn_{1.12}Al_1$	
**Element, oxidation state, form**	Composition (at. %)					
	calc	act	calc	act	calc	act
**Cu: Cu^0, Cu^+, Cu^{2+}, CuO, Cu_2O**	8.9	10.8	11.3	19.8	12.2	8.4
**Zn: ZnO, $Zn(OH)_2$, $ZnAl_2O_4$**	24.8	25.3	17.9	20.2	22.0	21.0
**Al: Al^{3+}, Al_2O_3, $Al(OH)_2$, AlOOH, $ZnAl_2O_4$**	38.4	40.1	46.9	38.3	43.4	48.7
**O: CuO, Cu_2O, ZnO, Al_2O_3, CO_3^{2-}**	24.4	21.1	19.5	18.4	20.3	17.7
**C: C-C/C-H, HCO_3^-/CO_3^{2-}**	3.5	2.7	3.1	3.1	2.1	2.8
**Cu/Zn**	0.36	0.43	0.63	0.98	0.55	0.40
**Cu/Al**	0.23	0.27	0.27	0.24	0.28	0.17
**Zn/Al**	0.65	0.63	0.38	0.53	0.50	0.43

Table S2. Distribution of acidic sites on reduced CuZnAl catalysts.

Catalyst	*Acidic site distribution ($mmol_{NH3}$ g^{-1})*			
	Weak sites	Moderate sites	Strong sites	Total acidity
$Cu_{0.61}$ $Zn_{1.26}$ Al_1	1.4	0.6	1.1	3.1
$Cu_{1.23}$ $Zn_{1.26}$ Al_1	2.3	0.8	1.2	4.3
$Cu_{2.28}$ $Zn_{1.12}$ Al_1	2.6	0.9	1.2	4.7

Table S3. Distribution of basic sites on CuZnAl catalysts.

Catalyst	*Basic site distribution ($mmol_{CO2}$ g^{-1})*			
	Weak sites	Moderate sites	Strong sites	Total basicity
$Cu_{0.61}$ $Zn_{1.26}$ Al_1	83.7	9.6	4.1	97.4
$Cu_{1.23}$ $Zn_{1.26}$ Al_1	35.3	29.7	8.5	73.5
$Cu_{2.28}$ $Zn_{1.12}$ Al_1	29.6	37.4	8.7	75.7

References

40. Zieliński, M.; Kaszkur, Z.; Juszczyk, W.; Sobczak, J. In Situ Diffraction Monitoring of Nanocrystals Structure Evolving during Catalytic Reaction at Their Surface. *Sci. Rep.* **2023**, *13*, 1469.
41. Beamson, G. High Resolution XPS of Organic Polymers. *Anal. Chim. Acta* **1993**, *276*, 469–470.
42. Chastain, J.; King Jr, R.C. Handbook of X-Ray Photoelectron Spectroscopy. *Perkin-Elmer Corp.* **1992**, *40*, 221.
43. NIST X-ray Photoelectron Spectroscopy Database (SRD 20); Version 5.0; National Institute of Standards and Technology: Gaithersburg, MD, USA, 2023. Available online: http://srdata.nist.gov/xps/ (accessed on 14 July 2024).
